# Physical activity, burnout and quality of life in medical students: A systematic review

**DOI:** 10.1111/tct.13525

**Published:** 2022-09-02

**Authors:** Charlotte E. Taylor, Emma J. Scott, Katherine Owen

**Affiliations:** ^1^ Warwick Medical School University of Warwick Coventry UK

## Abstract

**Background:**

Medical students are at risk of burnout and reduced quality of life (QoL). The risk of burnout doubles from third to sixth year of medical school, and medical students have an 8%–11% lower QoL than nonmedical students. It is imperative to prevent this, as burnout and reduced QoL is independently associated with errors in practice. This systematic review aims to examine whether physical activity/exercise is associated with burnout and/or QoL in medical students.

**Methods:**

Articles were identified through database searches of Embase, Medline, PsycINFO, Scopus and Web of Science. Studies were included if both physical activity/exercise and burnout or QoL were measured and limited to those focussing on medical students. Risk of bias was assessed using accredited cohort and cross‐sectional checklists. A narrative synthesis was conducted due to heterogeneity in the dataset.

**Findings:**

Eighteen studies were included, comprising 11,500 medical students across 13 countries. Physical activity was negatively associated with burnout and positively associated with QoL. Furthermore, the findings were suggestive of a dose–response effect of physical activity on both burnout and QoL; higher intensities and frequencies precipitated greater improvements in outcomes.

**Conclusions:**

This multinational review demonstrates that physical activity is associated with reduced burnout and improved QoL in medical students. It also identifies a paucity of research into the optimal intensity, frequency, volume and mode of physical activity. Further research, building on this review, is likely to inform the long overdue development of evidence‐based, well‐being curricula. This could involve incorporating physical activity into medical education which may improve well‐being and better prepare students for the demands of medical practice.

## 1 BACKGROUND

Burnout and quality of life (QoL) are two important concepts which affect well‐being.

Burnout is ‘a syndrome conceptualized as resulting from chronic workplace stress that has not been successfully managed’.^1^ There are three founding domains of burnout: emotional exhaustion (feelings of exhaustion due to stress), depersonalisation (distancing and impersonalising of one's work) and personal accomplishment (feelings of achievement and competence in work).^2^ The World Health Organisation defines QoL as ‘an individual's perception of their position in life in the context of the culture and value systems in which they live and in relation to their goals, expectations, standards and concerns’.^3^ Burnout and QoL are interrelated, with evidence of significant associations between QoL and all three domains of burnout.^4^

Recent findings show that 31.5% of UK doctors have ‘high burnout’.^5^ Furthermore, both burnout and reduced QoL in physicians have been independently associated with errors in practice and thus patient safety.^6^, ^7^, ^8^ Burnout has also been associated with reduced professional work effort in doctors.^9^

Medical students are particularly at risk of burnout and/or reduced QoL due to stressors experienced during training, including time pressure, coping with death and suffering, workload and maintaining work‐life balance.^10^ Medical students show an 8%–11% decreased QoL compared with non‐medical students of the same age.^11^ Additionally, the risk of burnout has been shown to double from third year to sixth year of medical school.^12^ Even small changes in burnout domains have been linked to a 7% increase in ‘serious thoughts’ of dropping out in the next year.^13^ This provides a strong impetus for improving well‐being strategies which students can use both in medical school and throughout their careers as doctors. There has been increasing interest in the integration of well‐being curricula to undergraduate medical education and more widely in health education^14^ with varying approaches such as mindfulness,^15^ lifestyle education^16^ and resilience training.^17^

Medical students are particularly at risk of burnout and/or reduced QoL due to stressors experienced during training.

One possible strategy is increasing medical students' levels of physical activity (‘any bodily movement produced by skeletal muscles that requires energy expenditure’^18^) and/or exercise (‘a subcategory of physical activity that is planned, structured, repetitive and purposeful in the sense that the improvement or maintenance of one or more components of physical fitness is the objective’^19^).

Indeed, physical activity has been linked to improvement in all domains of QoL,^20^ and an exercise programme in medical residents and fellows has been shown to significantly raise QoL.^21^ Furthermore, a systematic review of multiple professions demonstrated a negative relationship between physical activity and the key component of burnout, emotional exhaustion.^22^

To date, there has been no systematic review demonstrating the extent to which, if any, physical activity (including exercise) has an effect on burnout/QoL in medical students. Understanding this is an important step in informing future development of evidence‐based well‐being curricula.

Understanding this is an important step in informing future development of evidence‐based well‐being curricula.

### 1.1 Aim and objectives

This systematic review aims to provide clarity on whether physical activity/exercise has a role to play in the well‐being of medical students. Specifically,

1. Is physical activity/exercise associated with burnout in medical students?
2. Is physical activity/exercise associated with QoL in medical students?

## 2 METHODS

### 2.1 Protocol

The systematic review protocol was registered on PROSPERO (https://www.crd.york.ac.uk/prospero/display_record.php?ID=CRD42020182616). The PRISMA checklist has been followed in the review process.^23^

### 2.2 Eligibility criteria

Inclusion criteria are as follows:

- Medical students, defined as individuals enrolled in an undergraduate medicine degree course at a higher education institution
- Measurement of physical activity or exercise
- Measurement of burnout or QoL
- A comparison between physical activity/exercise level and burnout or QoL
- English language or studies with an English translation

Exclusion criteria are as follows:

- Medical students with vastly different curricula, for example, osteopathic, dental and preventative medicine
- Conference abstract only
- Letters, editorials, reviews and commentaries containing no new data
- Studies conducted during the COVID‐19 pandemic (due to differences in availability of physical activity/exercise)

### 2.3 Search strategy

Searches were conducted on Embase, Medline, PsycINFO, Scopus and Web of Science between 28 April 2020 and 1 May 2020 and updated on 21 February 2021. Key words used in the search were ‘medical students’ OR ‘undergraduate medical education’ OR ‘medical school’ OR ‘student doctors’ AND ‘physical activity’ OR ‘exercise’ AND ‘well‐being’ OR ‘well‐being’ OR ‘QoL’ OR ‘burnout’. No limits were placed on year of publication.

### 2.4 Study selection

Search results were exported into Endnote, reference management software. Titles and abstracts were screened to produce a shortlist according to the inclusion and exclusion criteria. Full texts were then obtained and reviewed to achieve a final list of studies for inclusion.

### 2.5 Data collection

Data extraction was completed using a pre‐designed extraction form. Author and date, country, number of participants, age, gender/sex, year of study, physical activity/exercise measure, burnout and/or QoL measure, other outcome measures and a summary of results were collected.

### 2.6 Risk of bias assessment

Cross‐sectional studies were critically appraised using the Center for Evidence‐Based Management (CEBMa) checklist.^24^ Cohort studies were critically appraised using the Critical Appraisal Skills Programme (CASP) cohort study checklist.^25^ Questions 7 and 12 referring to the results and implications for practice were removed from the CASP checklist; these could not be categorised for inclusion in the table however are considered in detail in the results and discussion of this review. An additional question referring to response rate was added to allow comparison to the cross‐sectional studies. No studies were excluded based on critical appraisal.

All stages of the review were undertaken by two authors (CT & ES) working in parallel. Any disagreements were resolved by discussion.

### 2.7 Synthesis of results

A narrative analysis was conducted for the main outcomes of burnout and QoL. Subgroups within the data were identified and analysed: pre‐clinical versus clinical students, physical activity/exercise intensity/volume/frequency/mode and questionnaire measures. Due to heterogeneity of study measures used and reported statistics, a meta‐analysis was not possible.

## 3 FINDINGS

### 3.1 Study selection

**FIGURE 1:**
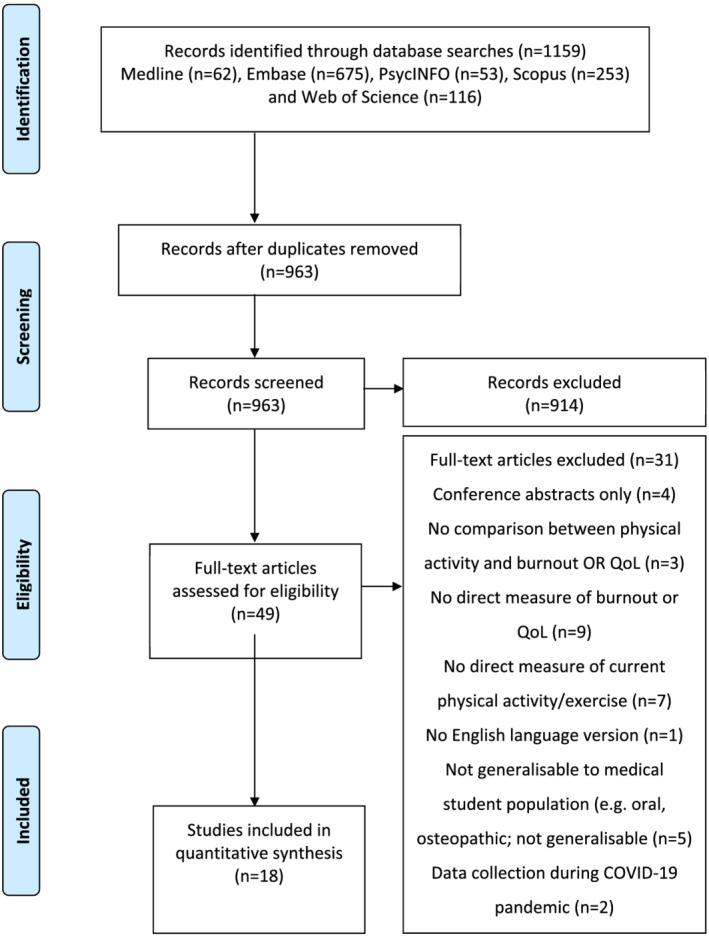
PRISMA flow diagram displaying results of database searches and screening

Figure 1 displays the results of the database searches. A total of 1159 articles were identified. After duplicates were removed, 963 studies remained which were screened by title and abstract. During this process, 914 papers were excluded. Reasons included the wrong population (e.g. non‐medical students), irrelevant topic and/or no mention of both physical activity/exercise and burnout/QoL. This left 49 studies for full‐text screening. Following this, 18 papers met the criteria and were included in the analysis.

### 3.2 Study characteristics

Of the 18 studies, 10 measured burnout, seven measured QoL and one measured both burnout and QoL. The search yielded a multi‐national selection; studies were carried out in Australia, Brazil, Canada, China, Hungary, Iran, Ireland, Lebanon, Saudi Arabia, Trinidad and Tobago, UK, USA, and Vietnam. The mean number of participants was 652 (Range: 54–4402). Of the studies that reported sex (*n =* 17), nine received more responses from females. The reported age range was 17–40 years. All the studies were observational; 17 were cross‐sectional and 1 was a cohort study.

### 3.3 Risk of bias assessment

Supporting information Tables S1 and S2 display the results of quality assessment including response rates. A 50% response rate was considered ‘good’. Overall quality of the studies was reasonable. An area of weakness for almost all cross‐sectional studies (*n =* 15) was the lack of a sample size based on considerations of statistical power. Several studies (*n =* 8) did not use confidence intervals for the results. The average response rate was 57%. Three studies had response rates of less than 30%.^26^, ^27^, ^28^

### 3.4 Measures

Physical activity/exercise was commonly measured with a questionnaire developed individually for the study (*n =* 11). Three used the Godin Leisure Time Exercise Questionnaire,^29^ three the International Physical Activity Questionnaire^30^ and one the Simple Lifestyle Indicator questionnaire.^31^ For transparency, the terminology used in each study has been transferred to this review (physical activity or exercise) in order to acknowledge the difference between the two terms.

The Maslach Burnout Inventory (MBI)^32^ or an adapted form of this, was the most common burnout measure (*n =* 9). Three used the MBI student survey,^33^ one the MBI general survey^32^ and one the MBI human services version.^32^ One used the Oldenburg Burnout Inventory student version^34^ and one used the Burnout Measure short version.^35^ For QoL, three used the SF‐36.^36^ Three used the WHO‐QOL‐Bref version.^37^ Two studies developed a questionnaire for their study. The VERAS‐Q^38^ was also used as an additional measure in one study.

### 3.5 Burnout and physical activity/exercise

Burnout findings are summarised in Table 1. Six studies^26^, ^39^, ^40^, ^41^, ^42^, ^43^ found a negative association between physical activity/exercise and all burnout components reported. Correlations ranged from −0.20 to −0.44; these are small‐to‐medium effect sizes.^44^ Three studies^27^, ^28^, ^45^ found that physical activity/exercise was associated with some burnout components but had no effect on others. Two studies^45^, ^47^ found no relationship between exercise and burnout.

**TABLE 1:** Summary of burnout studies included in analysis

Burnout studies				
Study and design	Numberof participants	Physical activity measure	Burnout measure	Results summary (comparison of physical activity and burnout)
Agarwal et al. (2020)^39^ Cross‐sectional	*n =* 287 Pre‐clinical students only	Lifestyle questionnaire recording amount of exercise	The Burnout Measure—short version	Burnout is negatively correlated with the amount of regular exercise (*r =* −0.272, *p =* 0.01) and exercise for stress relief (−0.292, *p =* 0.01).
Babenko et al. (2018)^40^ Cross‐sectional	*n =* 200	Godin Leisure Time Exercise Questionnaire	Oldenburg burnout inventory—student version (16 items)	Moderately strong negative correlation between leisure‐time exercise and exhaustion (−0.44, *p <* 0.01) Leisure‐time exercise also contributed to explaining exhaustion in the regression analysis (*B =* −0.12, *P =* 0.044) alongside psychological variables.
Bore et al. (2016)^26^ Cross‐sectional	*n =* 127	Questionnaire: hours of exercise and other physical activities in a typical week	MBI ‘Adapted for tertiary level students’ Same subscale structure as original MBI	Exercise is negatively correlated with burnout total (*r =* −0.29/−0.15). (correlation/standardised B‐value of significant unique predictor). Exercise is negatively correlated with lacking personal accomplishments (*r =* −0.22), emotional exhaustion (*r =* −0.30) and depersonalisation (*r =* −0.20). The burnout regression model had the largest number of significant unique predictors, including low exercise hours per week.
Cecil et al. (2014)^27^ Cross‐sectional	*n =* 356	IPAQ—short form. Low, moderate or high levels of physical activity. Metabolic equivalents (METs/week)	MBI	Physical activity was associated with higher personal accomplishment and lower emotional exhaustion. Low levels of physical activity in comparison to high levels (*B =* 0.15, *p =* 0.005), significantly predicted emotional exhaustion scores. *B =* 5.62 (1.99). 95% CI 1.69; 9.55. (Reference is high level of physical activity) Moderate (*B =* −0.12, *p =* 0.027) or low levels (*B =* −0.17, *p =* 0.002) of physical activity in comparison with high levels were significant predictors of lower personal accomplishment score. Physical activity was not related to depersonalisation scores.
Fares et al. (2016)^46^ Cross‐sectional	*n =* 165 Pre‐clinical students only	Questionnaire: Physical exercise is one category Yes/No reporting	MBI‐SS	The percentage of students with high emotional exhaustion (*p =* 0.794), high cynicism (*p =* 0.181), low academic efficacy (*p =* 0.075) or high burnout (*p =* 0.358) was not significantly different between those who engaged in exercise and those who did not.
Lee et al. (2020)^42^ Cross‐sectional	*n =* 731	Godin leisure‐time questionnaire	MBI	Participants who had burnout had lower exercise scores (*p =* 0.034). Participants who reported they ‘often’ did exercise were less likely to experience burnout than participants who reported ‘sometimes’ or ‘rarely’ (*P <* 0.001). Additionally, those who reported performing regular exercise were less likely to experience burnout than those who reported they ‘rarely’ exercised (*p =* 0.001). High emotional exhaustion was predicted by low reported exercise frequency (never/rarely versus often), *p <* 0.001.
Macilwraith et al. (2018)^45^ Cross‐sectional	*n =* 383	IPAQ‐short form	MBI‐SS	Weak positive correlation between professional efficacy and physical activity level (*r =* 0.134, *p =* 0.05). No significant correlation between emotional exhaustion and physical activity or personal accomplishment and physical activity.
Shadid et al. (2020)^47^ Cross‐sectional	*n =* 356	Questionnaire: Physical exercise is one category of extracurricular activities: Yes/No reporting	MBI‐SS	Exercise was not associated with emotional exhaustion, cynicism, academic efficiency or overall burnout (*p* > 0.05). Individuals who did sports had lower levels of burnout than those who did not do sports although this was not significant.
Wolf et al. (2017)^28^ Cohort (two time‐points)	*n =* 190 (first time) *n =* 149 (second time)	Godin Leisure Time Exercise Questionnaire	MBI‐GS	Those who exercised less had a higher risk of low professional efficacy. These participants also had a higher risk of exhaustion (*p =* 0.063), but this was not significant. Increase in activity level trended towards significance (*p =* 0.061) for predicting risk of burnout independently with the professional efficacy subscale (in the multivariate analysis). No association was found between leisure‐time exercise and cynicism. There was also no significant correlation between exercise and burnout.
Youssef et al. (2016)^43^ Cross‐sectional	*n =* 381 Pre‐clinical students only	Questionnaire with the statement ‘I exercise on a regular basis’ Yes/No reporting	MBI‐HSS	Students who exercised regularly showed reduced rates of burnout (*p =* 0.02).

Abbreviations: IPAQ, International Physical Activity Questionnaire; MBI, Maslach Burnout Inventory; MBI‐GS, General survey; MBI‐HSS, Human services survey; MBI‐SS, Student survey.

Nine of the 11 burnout studies directly measured emotional exhaustion, the key component of burnout.^2^ Of these, three^26^, ^39^, ^40^ found negative correlations between exercise and emotional exhaustion ranging from −0.272 to −0.44. Two^27^, ^42^ found that low levels of physical activity significantly predicted higher emotional exhaustion scores. However, four studies^28^, ^45^, ^46^, ^47^ did not find a significant relationship between physical activity/exercise and emotional exhaustion.

### 3.6 QoL and physical activity

QoL findings are summarised in Table 2. Six studies^40^, ^48^, ^49^, ^50^, ^51^, ^52^ found that physical activity/exercise improved all QoL domains measured, while two studies found that physical activity improved some, but not all, of the domains.^53^, ^54^

**TABLE 2:** Summary of QoL studies included in analysis

QoL studies (+ Dyrbye et al. with burnout and QoL)				
Study and design	Number of participants	Physical activity measure	Quality of life measure	Results summary (comparison of physical activity and QoL)
Ball et al. (2002)^48^ Cohort (four time‐points)	*n =* 54 Pre‐clinical students only	Health habits survey: frequency of exercise per month	Medical education QoL questionnaire	Satisfaction at midterm was predicted by a greater exercise frequency (*r =* 0.27, *p =* 0.05) No link between exercise and QoL was reported at any other time point.
Ghassab‐Abdollahi et al. (2020)^53^ Cross‐sectional	*n =* 186	IPAQ	WHO‐QOL BREF	Moderate physical activity was found to be a significant predictor of QoL (*p =* 0.016). Direct correlations were found between total score of QoL and subdomains with total physical activity (*p <* 0.05) except for the social relationship domain.
Jamali et al. (2013)^49^ Cross‐sectional	*n =* 1086	Questionnaire: frequency of >30 min of moderate physical activity. Daily, weekly (1–6 per week), occasionally (< once per week)	SF‐36	Students who were physically active daily had higher mean scores than those who were never active for physical component score (89.44 ± 11.11 versus 63.32 ± 20.90) and mental component score (83.48 ± 15.09 versus 48.01 ± 26.38), *p <* 0.001. Logistic regression showed that daily physical activity was significantly associated with a higher physical and mental component score after adjustment for variables (*p <* 0.001).
Lins et al. (2015)^54^ Cross‐sectional	*n =* 180	Questionnaire: regular physical activity, reported as yes/no	SF‐36	Lower mental component summary scores were significantly associated with a lack of regular physical activity (*p =* 0.018) Physical activity was not significantly associated with the physical component summary of the SF‐36 (*p =* 0.163)
Peleias et al. (2017)^50^ Cross‐sectional	*n =* 1350	Two questions: (a) ‘Which physical activity do you practice regularly?’ (b) ‘How many hours/week do you practice?’ Metabolic Equivalents (METs/week)	WHO‐QOL‐Bref VERAS‐Q	A significant association was found between physical activity (moderate/high levels) and better QoL for all components. For those with low levels of physical activity, this association was significant for *most* QoL measures (excluding physical health and social relationship domains). A strong positive relationship between the volume of physical activity and QoL was observed (dose–response effect). High‐volume physical activity was associated with improved scores in all the VERAS‐Q QoL domains.
Terebessy et al. (2016)^51^ Cross‐sectional	*n =* 629	Simple lifestyle indicator questionnaire	SF‐36	Regular vigorous exercise predicted improved mental health status (*F*(1) = 5.505, *p =* 0.019) compared with those who were less active.
Vo et al. (2020)^52^ Cross‐sectional	n = 712	Sociodemographic questionnaire: frequency of physical activity Never, 1–2, 3–4, >4 times/week	WHOQOL‐Bref	Those who were physically active 3–4 or 4 + times per week had significantly higher scores in 3 of the QoL domains and higher scores in the social domain although this was not significant: physical (*p =* 0.000), psychological (*p* = 0.000), environmental (*p =* 0;001), social (0.133). There was a significant dose–response effect in the physical, psychological and environmental domains.
Dyrbye et al. (2017)^41^ Cross‐sectional	*n =* 4402	Questionnaire: No. of minutes per week spent in moderate intensity, vigorous intensity and strength training	Burnout: MBI QoL: linear analogue scale from ‘As bad as it can be’ to ‘As good as it can be’. Score of ≥8 = high overall QoL.	Compliance with the CDC guidelines was independently associated with risk of burnout and QoL *after* controlling for confounders. The odds ratios for compliance with CDC guidelines versus not: Aerobic exercise and burnout OR = 0.79 (0.69,0.92 95% CI, *p =* 0.002) Strength training and burnout OR = 0.81 (0.75, 0.99 95% CI, *p =* 0.04) Aerobic exercise and QoL OR = 1.78 (1.56, 2.02 95% CI, *p <* 0.0001) Mean emotional exhaustion, depersonalisation and burnout were lower in students who met the aerobic exercise guidelines (*P <* 0.01). Mean emotional exhaustion score and prevalence of high emotional exhaustion and burnout were lower in those who met the CDC strength guidelines (*P <* 0.0001). These parameters were also lower for students who met both aerobic and strength guidelines. Mean QoL scores were higher for those following the CDC guidelines: Aerobic 7.2 versus 6.6 (*p <* 0.0001), Strength 7.2 versus 6.8 (*p <* 0.0001), Both 7.3 versus 6.8 (*p <* 0.0001).

Abbreviations: CDC, The Centres for Disease Control and Prevention; IPAQ, International Physical Activity Questionnaire; MBI, Maslach Burnout Inventory; SF‐36, 36‐item Short Form Survey; WHOQOL‐Bref, World Health Organisation Quality of Life‐brief version.

### 3.7 Pre‐clinical versus all med students

Four studies recruited pre‐clinical students only.^39^, ^43^, ^46^, ^48^ There were mixed results in both the pre‐clinical only and all medical student studies suggesting this was not an influential factor.

### 3.8 Physical activity/exercise intensity, volume, frequency and mode

Physical activity/exercise data were reported using a variety of categorical and continuous measures.

Three studies were categorised by exercise intensity.^27^, ^50^, ^51^ One study^51^ found that *only* vigorous exercise was associated with better QoL, compared with low and moderate intensity. A second study^27^ found that a *low* intensity of physical activity in comparison to high was a significant predictor of higher emotional exhaustion score. Additionally, moderate or low intensities of physical activity were significant predictors of a lower personal accomplishment score, whereas high levels were not.^27^ A third study^50^ found a significant association between both moderate and high levels of physical activity and better QoL for all components. Low activity level was also significantly associated with most QoL components, except physical health and social relationships.^50^

Six studies used a continuous variable for exercise.^26^, ^28^, ^39^, ^40^, ^45^, ^53^ Three of these studies^26^, ^39^, ^40^ found a negative association between increasing exercise and either exhaustion^40^ or total burnout.^26^, ^39^ Additionally, a *low* exercise level was a significant unique predictor of burnout,^26^ exhaustion and low professional efficacy.^28^ For QoL, significant correlations between total physical activity level and QoL (including all subdomains) were found.^53^ Additionally, moderate physical activity was a significant predictor of QoL.^53^ A second study found a weak positive correlation between professional efficacy and increasing physical activity levels. However, they found no correlation between increasing physical activity and emotional exhaustion or cynicism.^45^

Four studies recorded the frequency of physical activity/exercise without differentiating for intensity.^42^, ^48^, ^49^, ^52^ It was found that physical and mental component scores had a significant dose–response effect with scores increasing as frequency of physical activity increased.^49^ Additionally, greater physical activity frequencies (3–4/4 + per week) had significantly higher scores in all four domains of QoL.^52^ This was supported by another study which found that midterm QoL was predicted by greater exercise frequency.^48^ Students with burnout had lower exercise levels.^42^ Additionally, those who ‘often’ exercised were less likely to experience burnout than those who ‘rarely’ or ‘sometimes’ exercised.^41^

Only one study^41^ separated exercise by mode (aerobic, strength training, aerobic and strength training combination). All three types improved burnout and QoL outcomes. However, aerobic exercise improved *all* outcomes significantly, while strength training and aerobic/strength training were not significantly related to high depersonalization score.^41^

Four studies^43^, ^46^, ^47^, ^54^ recorded physical activity/exercise with a ‘yes/no’ or ‘agree/disagree’ method and therefore did not provide information on intensity, volume, frequency or mode.

## 4 DISCUSSION

### 4.1 Key findings

This systematic review aimed to discern whether physical activity/exercise is associated with burnout and/or QoL in medical students. The findings suggest that physical activity/exercise is associated with reduced burnout and increased QoL. Additionally, the data indicated that although all levels of physical activity/exercise can precipitate improvements in QoL and burnout, higher intensities and frequencies may be required for the greatest effect.

The findings suggest that physical activity/exercise is associated with reduced burnout and increased QoL.

Despite most studies supporting these findings, two studies^46^, ^47^ drew contradictory conclusions. This may have been due to method of exercise measurement; the studies used a binary ‘yes/no’ measure. Wide variation may have been present within the ‘yes’ category, ranging from walking to athlete training. This may explain the lack of a difference between yes and no groups. Although two other studies^43^, ^54^ also used dichotomous reporting styles, these were supportive of the consistent finding that physical activity/exercise reduced burnout. This may have been because they specified *regular* physical activity/exercise in their questions, diminishing this issue to some extent.

A wide range of tools were used to measure the outcomes of interest in this review. For example, the MBI is designed to assess the three dimensions of emotional exhaustion, depersonalisation and personal accomplishment.^55^ Adaptations have been developed; the MBI‐HSS is most applicable to human services jobs, such as the medical profession. Additionally, the MBI‐GS is applicable to roles without a large human service element, and the MBI‐SS is for students who are not in full‐time employment.^33^ The studies investigating burnout which found mixed results or no effect of physical activity used either the MBI‐GS or MBI‐SS.^28^, ^45^, ^46^, ^47^ It is plausible that this observation is due to the questionnaires being less applicable to the medical student population. Despite ‘student’ status, medical students spend a large proportion of time with patients in the future workplace.

Non‐MBI measures were also used, complicating results interpretation. The Burnout Measure is highly correlated with the emotional exhaustion subscale of the MBI,^56^ while the OBI has been validated^57^ as a measure of two dimensions: disengagement and exhaustion. The studies using these measures supported the majority findings so are unlikely to have been influenced by questionnaire choice.

Differences have also been identified in QoL questionnaires; the SF‐36 measures health‐related QoL, while the WHOQOL‐BREF measures global QoL.^58^ There was less discrepancy in results between studies using different questionnaires than for burnout studies suggesting that questionnaire choice was less of an issue for QoL studies.

This is the first review to specifically examine the effect of physical activity/exercise on burnout and/or QoL in medical students. Reviews in other populations found a negative correlation between physical activity and emotional exhaustion in employees from a mixture of professions.^22^ Additionally, individual studies in teachers^59^ and medical residents/fellows^21^ have found negative correlations between physical activity and burnout.

This is the first review to specifically examine the effect of physical activity/exercise on burnout and/or QoL in medical students.

There are contrasting reviews; a recent meta‐analysis of RCTs showed no significant difference in burnout between physical activity intervention and control groups in employees of various professions.^60^ However, this meta‐analysis only included four studies with a wide range of exercise modalities.^60^

Previous research of QoL is more limited. Surgeons who completed aerobic and strengthening exercises according to US guidelines had high QoL scores compared with those who did not meet the guidelines.^61^ Additionally, a study of University students (18–30 years; some from physical health/physiotherapy) found that physical activity was positively correlated to QoL.^62^ However, this was only for certain types of physical activity such as household tasks rather than leisure‐time physical activity, for which a relationship was not established. These studies support our findings; however, they must be treated with caution. More detail is required on the type of physical activity which precipitates improvements.

### 4.2 Strengths

This is the first review to examine the effect of physical activity/exercise on either burnout and/or QoL in medical students. It therefore provides a unique synthesis of information drawn from around the globe which has implications for medical schools worldwide. The consideration of both burnout and QoL allows for the opportunity to evaluate whether physical activity influenced both, one or neither of these interlinked concepts. Additionally, subgroups, such as physical activity/exercise intensity, have been identified and analysed in detail to draw further conclusions from the findings.

A further strength is the demographics of studies included. Over 11,500 students from all stages of medical school over 13 countries were included. This suggests that the improvement of QoL and reduction of burnout via physical activity demonstrated are not limited to a single medical curriculum or stage of study.

Over 11,500 students from all stages of medical school over 13 countries were included.

### 4.3 Limitations

The predominance of cross‐sectional study design is a limitation as without follow‐up, causality cannot be inferred. Reverse causality is plausible; those who were less burned‐out and had higher QoL may have had greater efficacy for physical activity/exercise. The only cohort study included in the analysis had a limited follow‐up duration of up to 1 year. Additionally, due to lack of data in some articles, if no relationship was reported between exercise and burnout/QoL, it has been assumed that there was no significant relationship. However, this may not be the case.

All studies were observational, and physical activity/exercise was self‐reported. This may have resulted in reporting bias. Despite attempts to standardise measures with metabolic equivalent multiplication in some studies, the initial number of bouts/minutes and intensity relies on participant recall.

There may also have been non‐response bias. The studies used opportunistic, voluntary samples with response rates ranging from 18% to 100%, with a mean of 57%. Participants who did not respond may have differed in burnout/QoL, and therefore, these may be under or over reported.

### 4.4 Future research and implications

There is a paucity of high methodological quality research in this area, demonstrated by the critical appraisal results. Future research should focus on longitudinal cohort and interventional studies to allow exploration of causality. More detail on physical activity/exercise is necessary; the intensity, frequency, volume and mode must be investigated. Additionally, a clearer distinction between physical activity and exercise is required, as this will influence recommendations made to medical schools. Sample sizes based on considerations of statistical power, homogeneity in burnout/QoL questionnaires, greater consideration of confounding factors and longer follow‐up in cohort studies will produce higher quality studies. Greater homogeneity of measures used will also allow for a future meta‐analysis. Importantly, future research must identify the minimum level of physical activity/exercise required to reduce burnout and increase QoL significantly. This would allow for maximal inclusivity when implemented into medical education.

Future research must identify the minimum level of physical activity/exercise required to reduce burnout and increase QoL significantly. This would allow for maximal inclusivity.

Medical admissions processes are being refined to identify students with ‘grit’^63^, ^64^ and the resilience to be able to complete a challenging programme of study.^65^ Evidence of sporting activity in applications is unlikely to be impactful compared with academic performance, admission tests and interviews, which is rightly supportive of applicants from widening participation backgrounds.^66^ National stakeholders^67^ emphasise the importance of facilitating access to medicine to students with disabilities and using physical activity as a factor in selection may be discriminatory. The authors therefore do not suggest that physical activity should be used as a selection criterion for medical school admissions.

Despite this opinion, the evidence presented in this systematic review leads the authors to propose that physical activity should be used as part of a spectrum of well‐being activities on offer during medical school. In this way, physical activity would not be compulsory for all medical students, rather, it could form part of a compulsory well‐being programme alongside other options, such as mindfulness.

Medical curricula are subject to repeated pressure to increase content on several fronts including sub‐specialisation of medical disciplines, medical advances, external stakeholder pressures and high‐profile patient safety events.^68^, ^69^ While there is increasing interest in well‐being curricula within medical schools, engagement from students is variable.^70^ As a course with high cognitive load, focus is maintained on credit‐bearing modules which are likely to be assessed in formal examinations. With crowded timetables students may be impeded from accessing their previous levels of activity; it is possible that a debate around required curriculum content for medical students is overdue. There is evidence that student engagement is higher with peer‐led or self‐directed well‐being activities^71^ and strategies which support this (in addition to freeing up time) such as reduced gym membership and support for student sports societies could be encouraged. Several US schools offer credit‐bearing modules for weight loss in obese students,^72^ and this approach could be applied with credits available for physical activity engagement.

If we are serious about facilitating learning in our students, we need to create a culture in which self‐care is valued and facilitated.^73^

Protected time for well‐being is a model which already exists in some educational activities in the UK. For example, many colleges and universities do not timetable learning on Wednesday afternoon to allow students to pursue interests of their choice, often sport. This could be successfully extended to medical education. Importantly, the authors suggest that well‐being activities should be given specific time in the timetable rather than expecting students to find time outside of medical education. It would also be interesting to investigate how these well‐being activities are best delivered; the COVID‐19 pandemic has mandated the use of online technology—could a physical activity intervention be successfully delivered in this way?

Well‐being activities should be given specific time in the timetable rather than expecting students to find time outside of medical education.

Additionally, it is imperative that medical students are educated about the importance of physical activity for both physical and mental health. By doing so, they will be equipped to offer advice to their patients and are more likely to understand the benefits for themselves.

It is pivotal that strategies are taken to improve well‐being in medical school, especially at a time where medical students are facing the unique challenges that the COVID‐19 pandemic has imposed. The authors believe that this systematic review provides an exciting basis for the inclusion of physical activity into well‐being measures during medical school.

## 5 CONCLUSION

This multinational systematic review demonstrates that physical activity is associated with lower burnout and increased QoL in medical students. Following further research addressing the limitations identified in this review, medical schools could prioritise well‐being of medical students via the implementation of tailored physical activity. This is of great significance for medical education and beyond, as the next generation of doctors may be better prepared to manage the demands of medical practice and offer optimal care to their patients.

## CONFLICT OF INTEREST

The authors have no conflict of interest to disclose.

## ETHICS STATEMENT

The authors have no ethical statement to declare.

## Supporting information


**Table S1:** Summary of critical appraisal for cross‐sectional studies
**Table S2:** Summary of critical appraisal for cohort studies
