# Supplementary material for: Physical activity, burnout and quality of life in medical students: A systematic review
Source: Clin Teach. 2022 Sep 2;19(6):e13525. doi: 10.1111/tct.13525 (PMC9826463; doi:10.1111/tct.13525)
Supplement: Supplementary file 1 — Table S1: Summary of critical appraisal for cross‐sectional studies Table S2: Summary of critical appraisal for cohort studies [file TCT-19-0-s001.pdf]

Supplementary table 1: Summary of critical appraisal for cross-sectional studies

First author (year)	Did the study address a clearly focused question/issue?	Is the research method (study design) appropriate for answering the research question?	Is the method of selection of the subjects (employees, teams, organizations) clearly described?	Did the way the sample was obtained minimise (selection) bias?	Were the subjects representative with regard to the population to which the findings will be referred?	Was the sample size based on pre-study considerations of statistical power?	Was a satisfactory response rate achieved? (%)	Are the measurements (questionnaires) likely to be valid and reliable?	Was the statistical significance assessed?	Are confidence intervals given for the main results?	Were relevant confounding factors identified and accounted for?	Can the results be applied to your organisation?
Agarwal (2020)	Green	Green	Orange	Green	Green	Red	86.7 (Green)	Green	Green	Red	Red	Green
Babenko (2018)	Green	Green	Red	Orange	Green	Red	NR (Orange)	Green	Green	Red	Red	Green
Bore (2016)	Green	Green	Green	Green	Orange	Red	18 (Red)	Green	Green	Green	Green	Green
Cecil (2014)	Green	Green	Green	Green	Green	Red	18.9 (Red)	Green	Green	Green	Red	Green
Dyrbye (2017)	Green	Green	Green	Green	Green	Red	35.2 (Red)	Green	Green	Green	Green	Green
Fares (2016)	Green	Green	Green	Green	Green	Red	100 (Green)	Green	Green	Green	Green	Green
Ghassab-Abdollahi (2020)	Green	Green	Green	Green	Green	Green	100 (Green)	Green	Green	Green	Green	Green
Jamali (2013)	Green	Green	Green	Green	Green	Red	80.4 (Green)	Green	Green	Green	Green	Green
Lee (2020)	Green	Green	Green	Green	Green	Red	55.6 (Green)	Green	Green	Orange	Green	Green
Lins (2015)	Green	Green	Green	Green	Green	Red	100 (Green)	Green	Green	Red	Green	Green
Macilwraith (2018)	Green	Green	Green	Green	Green	Red	38.2 (Red)	Green	Green	Red	Green	Green
Peleias (2017)	Green	Green	Green	Green	Green	Green	81.8 (Green)	Green	Green	Green	Green	Green
Shadid (2020)	Green	Green	Green	Green	Green	Red	71.2 (Green)	Orange	Green	Green	Green	Green
Terebessy (2016)	Green	Green	Green	Green	Green	Red	69.8 (Green)	Green	Green	Green	Green	Green
Vo (2020)	Green	Green	Green	Green	Green	Red	NR (Orange)	Green	Green	Red	Green	Green
Wolf (2017)	Green	Green	Green	Green	Orange	Red	28.7 (Red)	Green	Green	Red	Green	Green
Youssef (2016)	Green	Green	Green	Green	Green	Red	85 (Green)	Green	Green	Red	Green	Green

Green = Yes, Orange = Indeterminate, Red = No. NR = Not reported

Supplementary table 2: Summary of critical appraisal for cohort studies

First author (year)	Did the study address a clearly focused issue?	Was the cohort recruited in an acceptable way?	Was the exposure accurately measured to minimise bias?	Was the outcome accurately measured to minimise bias?	Was a satisfactory response rate achieved? (%)	Have the authors identified all the confounding factors in the design and/or analysis?	Was the follow up of subjects complete enough?	Was the follow up of subjects long enough?	How precise are the results e.g. confidence intervals	Do you believe the results?	Can the results be applied to the local population?	Do the results of this study fit with other available evidence?
Ball (2002)	[Green]	[Green]	[Green]	[Orange]	NR [Orange]	[Green]	[Green]	[Green]	[Red]	[Green]	[Green]	[Green]

Green = Yes, Orange = Indeterminate, Red = No. NR = Not reported.
